# Osteopontin, osteoprotegerin and musculoskeletal ultrasound findings in first-degree relatives of rheumatoid arthritis: potential markers of preclinical disease

**DOI:** 10.1186/s12891-024-07291-7

**Published:** 2024-03-05

**Authors:** Eiman Soliman, Sarah Ohrndorf, Magdy Zehairy, Khaled Matrawy, Abeer Alhadidy, Abeer Abdelati

**Affiliations:** 1 Internal Medicine Department, Rheumatology and Clinical Immunology Unit, Faculty of Medicine, Alexandria University, Alexandria, Egypt; 2 Department of Rheumatology and Clinical Immunology, Charité-Universitätsmedizin Berlin, 10117 Berlin, Germany; 3 Radiodiagnosis Department, Medical Research Institute, Alexandria University, Alexandria, Egypt; 4 Clinical Pathology Department, Faculty of Medicine, Alexandria University, Alexandria, Egypt

**Keywords:** Osteopontin, Osteoprotegerin, Ultrasound, First degree relatives, Rheumatoid

## Abstract

**Background:**

First-degree relatives (FDRs) of rheumatoid arthritis (RA) patients are known to have increased risk of developing the disease. The detection of altered bone metabolism in FDRs could be a predictor of the disease. Musculoskeletal ultrasound (MSUS) is known for its ability to detect subclinical joint inflammation in RA, but changes in FDRs are not yet described. We aimed to study serum Osteopontin (OPN) and Osteoprotegerin (OPG) levels in FDRs of RA patients as markers of altered bone metabolism in relation to clinical, laboratory and musculoskeletal ultrasound (MSUS) findings.

**Methods:**

Fifty-five individuals were included, 20 had definite RA, 25 were first degree relatives (FDRs) of RA patients, and 10 healthy controls. Clinical evaluation for joint swelling/tenderness was performed for all. ESR, CRP, rheumatoid factor (RF), anti-citrullinated antibodies (ACPA), OPN, OPG, and Musculoskeletal ultrasound (MSUS) by the US7 score were evaluated.

**Results:**

Osteoprotegerin was significantly higher in RA (143.89 pg/ml ± 365.47) than in FDRs (22.23 pg/ml ± 65.73; *p* = 0.009) and controls (6.20 pg/ml ± 12.43; *p* = 0.003). OPN was also higher in RA (3.66 ng/ml ± 4.20) than in FDRs (1.97 ng/ml ± 1.04) and controls (2.81 ng/ml ± 1.31), though not significant (*p* = 0.102). Eight of 25 FDRs (32%) had arthralgia without clinical arthritis and 17/25 (68%) were asymptomatic. FDRs with arthralgia had significantly higher ESR and CRP levels than asymptomatic FDRs (9.82 mm/h ± 4.13; *p* = 0.003, and 3.93 mg/l ± 3.58; *p* = 0.003). Osteoprotegerin was higher in FDRs than in controls, and also in those with arthralgia (51.55 pg/ml ± 114.68) than in those without (8.44 pg/ml ± 9.67), though without significant difference. OPN was higher in FDRs with arthralgia (2.09 ng/ml ± 1.19) than in asymptomatic (1.70 ng/ml ± 0.55), also without significant difference. Pathologic findings by US7 were detected in 10/25 (40%) FDRs, of which three (12%) had arthralgia and seven (28%) were asymptomatic.

**Conclusions:**

The raised OPG and lower OPN in FDRs than in controls reflect an altered bone metabolism which could precede clinical disease phase. OPN and OPG could serve as markers of altered preclinical bone metabolism in FDRs of RA. US7 score might be a useful screening tool to identify ‘at-risk’ individuals.

**Supplementary Information:**

The online version contains supplementary material available at 10.1186/s12891-024-07291-7.

## Background

Rheumatoid arthritis (RA) is a chronic inflammatory autoimmune disease that commonly leads to progressive joint damage, impaired function, and progressive disability [1]. Bone resorption is a hallmark of RA; however, bone destruction may occur before the detection of inflammation in the joints of anti-citrullinated peptide protein antibody (ACPA) positive individuals at risk for RA not yet having the clinical evident disease [2, 3]. The increased rate of bone loss in RA patients is referred to several disease-related and patient-related factors. The effect of chronic inflammation of RA with overproduction of multiple pro-inflammatory cytokines play the major role in the development of osteoporosis and altered bone metabolism in RA patients [4].

A positive family history of RA increases the risk of RA in 3 to 9 times [5]. Higher levels of multiple cytokines and chemokines were found to be associated with RA-related autoantibody positivity in first degree relatives (FDRs) without clinically apparent RA. Accordingly, FDRs can represent the preclinical phase of RA [6].

Early diagnosis of RA is important because early treatment reduces the long-term disability. Clinical examination and laboratory tests are limited for early detection of subclinical synovitis. Conventional radiography is insensitive in detecting early bone damage [7]. Musculoskeletal ultrasound (MSUS) including power Doppler (PD) is increasingly used to evaluate joint involvement in RA, particularly early and subclinical synovitis, due to its high sensitivity in depicting local inflammation such as synovitis and tenosynovitis [8].

Osteopontin (OPN) works as a proinflammatory cytokine that can modulate the immune response by enhancing expression of Th1 cytokines and matrix degrading enzymes [9]. Increased expression of OPN is found in RA [10]. The involvement of OPN in RA has been explained by OPN-induced migration, differentiation, and functional activation of osteoclasts (OCs) and T-lymphocytes [11], leading to pathological processes of RA [12].

Osteoprotegerin (OPG) regulates bone resorption mainly by inhibiting osteoclastic bone resorption and also promoting osteoclast apoptosis [13]. OPG works as a decoy receptor, preventing association of receptor activator of nuclear factor-kappa B ligand (RANKL) with RANK receptor [14, 15], and thus moderating osteoclastogenesis and bone resorption. An imbalance of this system may be partly responsible for the skeletal complications of RA [16].

FDRs of RA are known to have increased risk of developing the disease [6]. The detection of altered bone metabolism in FDRs could be a predictor of the disease. Preclinical phase of RA is also characterized by a state of autoimmunity and inflammation [17].

MSUS is known for its ability to detect subclinical synovitis, [18] but changes in first FDRs are not yet described.

The aim of our work was to study OPN and OPG, as markers of altered bone metabolism in FDRs of RA patients in relation to clinical manifestations, laboratory markers, and MSUS findings in FDRs compared to RA patients and healthy individuals. Furthermore, FDRs with and without clinical symptoms (arthralgia) were compared.

## Methods

This observational study was conducted between December 2018 and February 2020 and included 55 persons divided into 20 RA patients with age above 18 and fulfilling the 2010 RA ACR/ EULAR criteria [19] (group І) and 25 FDRs of RA patients (group II). First degree relatives with past or present arthritis (defined as one or more swollen joints), age < 18 years, previous treatment with a disease-modifying anti-rheumatic drug or recent glucocorticoid treatment, systemic autoimmune disease, systemic infections, lymphoproliferative disorders were excluded from the study. Ten healthy controls (group III) with matched age and sex were also included.

All participants gave their informed consent. The study was approved by the local ethical committee (no. 20/179)before initiation of the study.

### Clinical assessment

All participants were subjected to full medical history including musculoskeletal and extra-musculoskeletal symptoms, comorbidities, medication history, past history, and family history. Complete clinical examination was performed with emphasis on details of joint manifestations. In RA patients, clinical assessment (tender/swollen joint count) was done using disease activity score DAS28(ESR), patient’s global assessment (PGA) and pain visual analogue scales (VAS 0-100 mm each) [20–22]. Also, the health assessment questionnaire (HAQ) was evaluated.

### Lab assessment

Laboratory investigations were performed to all groups and included erythrocyte sedimentation rate (ESR; normal level < 10 mm/h), C-reactive protein (CRP; normal level < 3.0 mg/l) [23], IgM-rheumatoid factor (RF) (normal level < 15.9 IU/ml) and anti-citrullinated protein antibodies (ACPA) (normal level < 20 U/ml) [24], serum levels of osteopontin (OPN) (ng/ml) [25] and osteoprotegerin (OPG) (pg/ml) [26].

### Imaging assessment

All RA patients and FDRs were scanned by an experienced EULAR certified MSUS trainer using the US7 score [27]. US7 score was conducted on the following joints of the clinically most affected hand and forefoot: wrist, MCP II, III, PIP II, III, MTP II, and V which were assessed for synovitis, tenosynovitis/paratenonitis, and erosions. Synovitis and synovial/tenosynovial vascularity were scored semiquantitatively (grade 0–3) on GS and PDUS. Tenosynovitis as well as erosions were scored for their presence (0/1). The wrist was examined in the dorsomedian, ulnar, and palmar aspects for synovitis and tenosynovitis in GS and PDUS, and for erosions. The MCP II and III were assessed in the dorsal aspect for synovitis in PDUS, for paratenonitis in GS and PDUS, and also for erosions. The radial aspect of MCP II was scanned for erosions, and in the palmar aspect for synovitis and tenosynovitis in GS and PDUS as well as for erosions. PIP joints II and III were examined in the dorsal aspect for synovitis in PDUS and for erosions, and in the palmar aspect for synovitis in GS and PDUS and for erosions. The toe joints MTP II and V were examined in the dorsal aspect for synovitis in GS and PDUS, and for erosions and in the plantar and lateral (only MTP V) aspects for erosions.

Sum scores for synovitis, tenosynovitis/paratenonitis, and erosions were calculated. The scoring for GS synovitis ranged from 0 to 27, for the PD synovitis score 0–39, for the GS tenosynovitis score 0–7, for the PD tenosynovitis score 0–21, for the erosions score 0–17 including wrist examination.

In asymptomatic FDRs the right hand and forefoot were examined (dominant side).

US examination of each patient took 20–30 min, including documentation.

Clinical evaluation and MSUS scanning were carried out on the same day of the blood sampling.

### Machine data

All scans were performed with the Acuson X150 Antares ultrasound system, premium edition (Siemens, Malvern, PA, USA) using linear array transducers VF 13 − 5 SP for finger and toe joints, operating at 11.43 MHz for GS and 8.9 MHz for PD.

### Conventional radiography

Plain x-rays of the hands and forefeet (of the MSUS scanned side) were available only for RA patients, but not for FDRs or controls. The presence of erosions was determined as present or absent (0/1).

### Statistical analysis of the data

Data were analyzed using IBM SPSS version 20 [28]. Qualitative data were described using number and percentage. Quantitative data were described using mean ± standard deviation, median and range (minimum and maximum). Significance of the obtained results was judged at the 5% level. The used tests were: *Chi-square test* for categorical variables to compare between different groups, *Fisher’s Exact or Monte Carlo correction* (correction for chi-square when more than 20% of the cells have expected count less than 5); *Student t-test* for normally quantitative variables to compare between two studied groups, *F-test (ANOVA)* for normally quantitative variables to compare between more than two groups, and Post Hoc test (LSD) for pairwise comparisons, *Kruskal Wallis test* for abnormally quantitative variables to compare between more than two studied groups, *Mann Whitney test* or abnormally quantitative variables to compare between two studied groups, and *Spearman coefficient* to correlate between two abnormally quantitative variables.

## Results

### Demographic and clinical data

Mean age in RA was 39.50 ± 13.43 years with a mean disease duration of 11.95 ± 8.36 years. In FDRs the mean age was 33.1 ± 13.4 years, and in controls it was 33.70 ± 6.99 years. In RA patients, 14 (70.0%) were female. In FDRs of RA patients, 19 (76.0%) were female. In healthy controls, six were (60.0%) female. There was no statistically significant difference between the three studied groups regarding age and sex.

All RA patients fulfilled the 2010 ACR/EULAR classification criteria [18] and were suffering from arthralgia/arthritis mainly symmetrical and polyarticular; the most commonly involved joints were the small joints of hands and feet. There were 17 (68%) of FDRs without arthralgia and eight (32%) with arthralgia (as arthritis was an exclusion criterion) that involved different joints of the hands, feet, knees and shoulders. None of the controls had current/past joint symptoms.

70% of RA patients had a disease duration of > 5 years, 90% showed high disease activity (DAS28 > 5.1), 70% had severe to very severe disability (HAQ score ≥ 2 to ≤ 3), and 80% presented with severe degree of fatigue (PGA > 50–100 mm) and severe pain (70–100 mm).

### Lab results

#### ESR and CRP

Mean ESR was significantly higher in RA (64.15 ± 34.29) than in FDRs (15.6 ± 11.04; *p* < 0.001) and controls (6.0 ± 2.05, *p* < 0.001) and significantly higher in FDRs than in controls (*p* = 0.001).

Mean CRP was significantly higher in RA (26.38 ± 29.14) than in FDRs (5.99 ± 5.08, *p* < 0.001) and controls (2.02 ± 0.53, *p* < 0.001) and significantly higher in FDRs than in controls (*p* = 0.011).

#### RF and ACPA

Mean RF and ACPA were statistically higher in RA than in FDRs and controls. Mean ACPA was higher in FDRs than in controls while there was no difference regarding mean RF between FDRs and controls. (Table 1)

**Table 1:** Rheumatoid factor and anti-citrullinated protein antibodies in the studied groups

	RA (*n* = 20)	FDRs (*n* = 25)	Controls (*n* = 10)	P	
**RF (< 15.9 IU/ml)**					
Negative No. (%)	2 (10.0%)	25 (100.0%)	10 (100.0%)	< 0.001^*^	
Positive No. (%)	18 (90.0%)	0 (0.0%)	0 (0.0%)		
Min. – Max.	9.0–697.0	4.80–12.70	8.20–11.75	< 0.001^*^	
Mean ± SD.	133.28 ± 176.38	9.21 ± 1.9	9.92 ± 1.19		
Median	68.40	9.19	9.40		
**Significance between groups**	p_1_ < 0.001^*^, p_2_ < 0.001^*^, p_3_ = 0.358				
**ACPA(< 20 U/ml)**					
Negative No. (%)	3(15%)	25(100%)	10(100%)	< 0.001^*^	
Positive No. (%)	17(85%)	0 (0%)	0 (0%)		
Min. – Max.	7.0–915.0	5.0–17.60	6.50–10.70	< 0.001^*^	
Mean ± SD.	226.83 ± 266.19	11.06 ± 3.43	9.53 ± 1.34		
Median	107.0	11.20	9.75		
**Significance between groups**	p_1_ < 0.001^*^, p_2_ < 0.001^*^, p_3_ = 0.131				

p value for Kruskal Wallis test for comparing between the different groups; Significance between groups was done using Mann Whitney test; p_1_: p value for comparing between RA patients and FDRs; p_2_: p value for comparing between RA patients and controls; p_3_: p value for comparing between FDRs and controls; *: Statistically significant at *p* ≤ 0.05

RA: rheumatoid arthritis; FDRs: first degree relatives; RF: rheumatoid factor; ACPA: anti-citrullinated protein antibodies

#### OPN and OPG

OPN was non-significantly higher in RA (3.66 ± 4.20) than in FDRs (1.97 ± 1.04) and controls (2.81 ± 1.31, *p* = 0.102). OPG was significantly higher in RA (143.89 ± 365.47) than in both FDRs (22.23 ± 65.73, *p* = 0.009) and controls (6.20 ± 12.43, *p* = 0.003). (Table 2)

**Table 2:** Serum Osteopontin and Osteoprotegerin in the studied groups

	RA (*n* = 20)	FDRs (*n* = 25)	Controls (*n* = 10)	P
**Serum OPN (ng/ml)**				
Min. – Max.	0.0–18.40	0.90–5.80	1.40–5.40	0.102
Mean ± SD.	3.66 ± 4.20	1.97 ± 1.04	2.81 ± 1.31	
Median	2.50	1.80	2.65	
**Serum OPG (pg/ml)**				
Min. – Max.	0.0–1558.0	0.0–334.1	0.0–39.80	0.004^*^
Mean ± SD.	143.89 ± 365.47	22.23 ± 65.73	6.20 ± 12.43	
Median	21.20	7.70	1.50	
**Significance between groups**	p_1_ = 0.009^*^, p_2_ = 0.003^*^, p_3_ = 0.332			

p value for Kruskal Wallis test for comparing between the different groups; Significance between groups was done using Mann Whitney test; p_1_: p value for comparing between RA patients and FDRs; p_2_: p value for comparing between RA patients and controls; p_3_: p value for comparing between FDRs and controls; *: Statistically significant at *p* ≤ 0.05

RA: rheumatoid arthritis; FDRs: first degree relatives; OPN: Osteopontin; OPG: Osteoprotegerin

Whereas, FDRs had higher mean OPG and lower OPN compared to controls but without reaching statistical significance. (Table 2)

In RA, mean OPN and OPG were higher in RF and ACPA positive RA than RF and ACPA negative patients but without reaching statistical difference. (Table 3)

**Table 3:** Serum Osteopontin and Osteoprotegerin in relation to different parameters in RA (*n* = 20)

	**(n = 20)**	OPG	**P**	OPN	**P**
		**Mean ± SD.**		**Mean ± SD.**	
**RF (< 15.9IU/ml)**					
Negative	2	60.50 ± 85.56	0.753	2.65 ± 0.35	0.753
Positive	18	153.15 ± 384.64		3.77 ± 4.43	
**ACPA (< 20U/ml)**					
Negative	3	42.47 ± 68.09	0.341	3.58 ± 2.58	0.289
Positive	17	161.78 ± 394.67		4.13 ± 3.48	
**Pain-VAS(0-10 mm)**					
5 - <7	4	14.58 ± 8.43	0.257	2.78 ± 2.39	0.344
7–10	16	176.21 ± 404.48		7.18 ± 7.92	
**PGA(0-100 mm)**					
0–50	4	14.58 ± 8.43	0.257	2.78 ± 2.39	0.344
> 50–100	16	176.21 ± 404.48		7.18 ± 7.92	

p values for Mann Whitney test for comparing between the two groups

OPN: Osteopontin; OPG: Osteoprotegerin; RF: rheumatoid factor; ACPA: anti-citrullinated protein antibodies; VAS: Visual Analogue Scale; PGA: Patient global assessment

In the three studied groups, no correlation was found between the measured markers of bone metabolism (OPN and OPG) and the different clinical and lab parameters (supplementary data).

#### Lab results in FDRs with and without arthralgia

Eight FDRs (32%) had arthralgia and 17 (68%) FDRs were asymptomatic. FDRs with arthralgia had significantly higher ESR and CRP values than asymptomatic FDRs (*p* = 0.003) (Table 4).

**Table 4:** Relation between articular manifestations and different parameters in first degree relatives (*n* = 25)

	Asymptomatic FDRs (*n* = 17)	FDRs with arthralgia (*n* = 8)	P
	No. (%)	No. (%)	
**ESR 1st hr(10 mm/hr)**			
Min. – Max.	5.0–20.0	9.0–45.0	0.003^*^
Mean ± SD.	9.82 ± 4.13	27.88 ± 11.22	
Median	8.0	27.50	
**CRP (< 3 mg/L)**			
Min. – Max.	0.82–13.0	2.88–19.0	0.003^*^
Mean ± SD.	3.93 ± 3.58	10.36 ± 5.21	
Median	2.78	9.87	
**RF (< 15.9 IU/ml)**			
Negative	17 (100)	8 (100)	-
Positive	0 (0)	0 (0)	
Min. – Max.	4.90–12.70	4.80–12.50	0.137
Mean ± SD.	8.55 ± 2.49	9.52 ± 1.54	
Median	9.18	9.19	
**ACPA (< 20 U/ml)**			
Negative	17 (100)	8 (100)	-
Positive	0 (0)	0 (0)	
Min. – Max.	7.40–17.0	5.0–17.60	0.560
Mean ± SD.	10.44 ± 3.31	11.35 ± 3.55	
Median	9.80	11.20	
**Serum OPN (ng/ml)**			
Min. – Max.	1.0–2.40	0.90–5.80	0.620
Mean ± SD.	1.70 ± 0.55	2.09 ± 1.19	
Median	1.60	1.80	
**Serum OPG (pg/ml)**			
Min. – Max.	0.0–27.20	0.0–334.10	0.314
Mean ± SD.	8.44 ± 9.67	51.55 ± 114.68	
Median	1.50	14.25	

p: p values for Mann Whitney test for comparing between the two groups; *: Statistically significant at *p* ≤ 0.05

FDRs: first degree relatives; ESR: erythrocyte sedimentation rate; CRP: c-reactive protein; OPN: Osteopontin; OPG: Osteoprotegerin

OPG was higher in FDRs than in controls (Table 2) and higher in those with arthralgia than in those without (n.s.; *p* = 0.314). Similarly, serum OPN was higher in FDRs with arthralgia than in asymptomatic FDRs (n.s.; *p* = 0.620). Furthermore, mean RF and ACPA were higher in FDRs with arthralgia than in FDRs without. (Table 4).

### MSUS findings

**Fig. 1:**
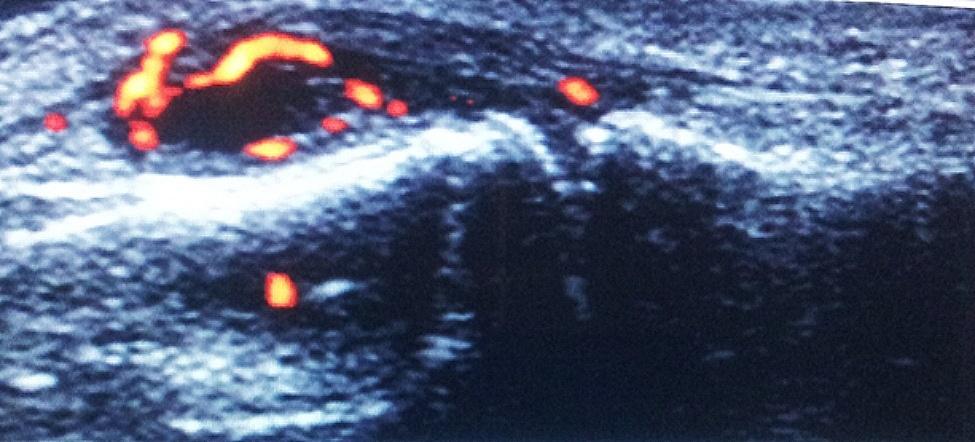
Dorsal longitudinal US scan of 2nd PIP joint showing synovitis with PD signal grade 3 in RA patient

**Fig. 2:**
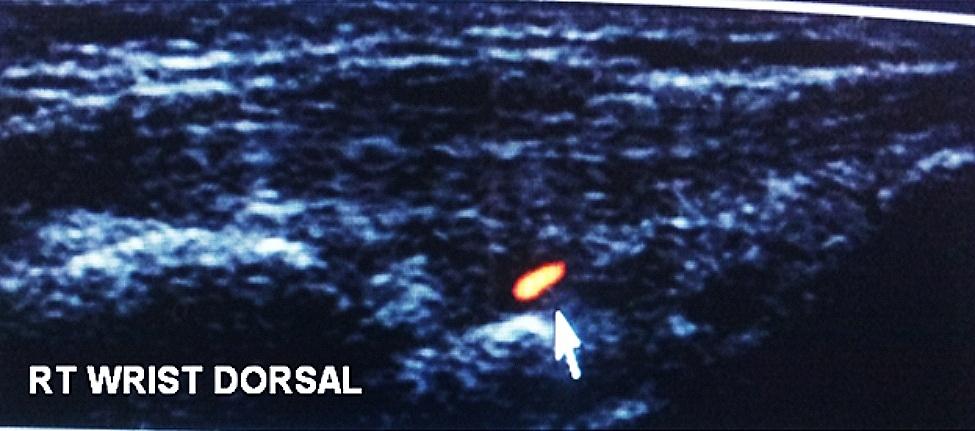
Dorsal longitudinal US scan of right (RT) wrist showing mild radiocarpal synovitis with PD signal grade 1 in a FDR

**Fig. 3:**
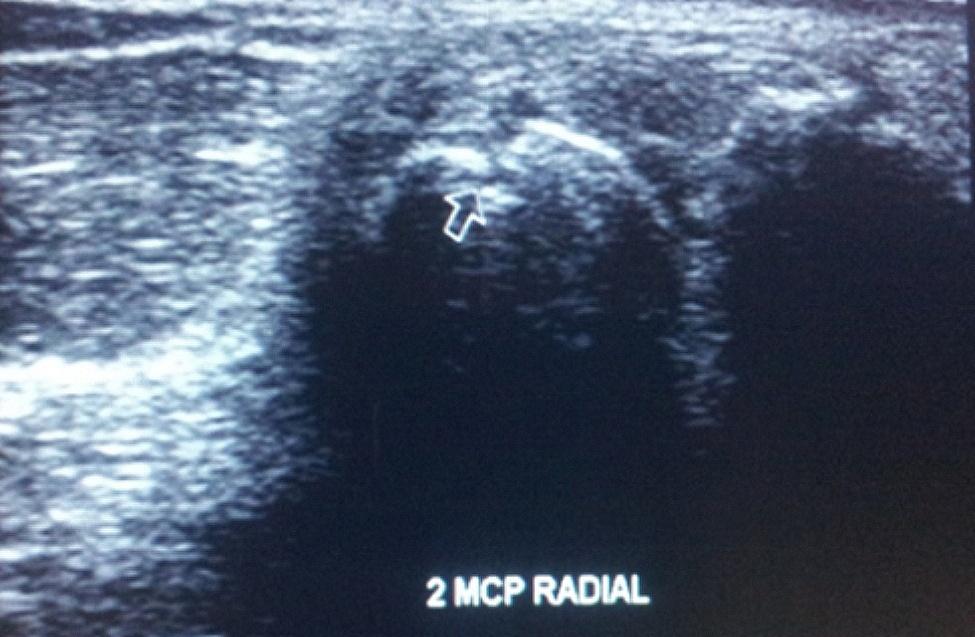
Radial longitudinal US scan of 2nd MCP joint showing erosion in a FDR

MSUS findings were detected in 16 (80%) RA and in ten (40%) FDRs of whom three FDRs (12%) had arthralgia and seven (28%) were asymptomatic. Synovitis was the commonest MSUS finding in RA (*n* = 16; 80%) and FDRs (*n* = 10; 40%) (Figs. 1 and 2). Tenosynovitis was present in five (25%) RA patients and in one (4%) FDR. Erosions were detected in nine (45%) RA patients and in one (4%) FDR (on radial MCP II scan) (Fig. 3) The comparison between rheumatoid arthritis patients and first degree relatives as regard 7-joint ultrasound score is shown in Table 5.

**Table 5:** Seven-joint ultrasound score in rheumatoid arthritis patients and first degree relatives

US7 score	RA (*n* = 20)	FDRs (*n* = 25)	P
Synovitis GS	5.15 ± 4.26	0.64 ± 1.04	< 0.001^*^
Synovitis PD	2.05 ± 2.54	0.24 ± 0.83	0.003^*^
Tenosynovitis GS	0.35 ± 0.75	0.04 ± 0.20	0.040^*^
Tenosynovitis PD	0.30 ± 0.80	0.0 ± 0.0	0.048^*^
Erosions	1.60 ± 3.10	0.04 ± 0.20	0.001^*^
Total score	9.45 ± 7.21	0.96 ± 2.01	< 0.001^*^

Showed values are mean ± SD; p: p values for Mann Whitney test for comparing between the two groups; *: Statistically significant at *p* ≤ 0.05

RA: rheumatoid arthritis; FDRs: first degree relatives; GS: Grayscale; PD: power Doppler

**Fig. 4:**
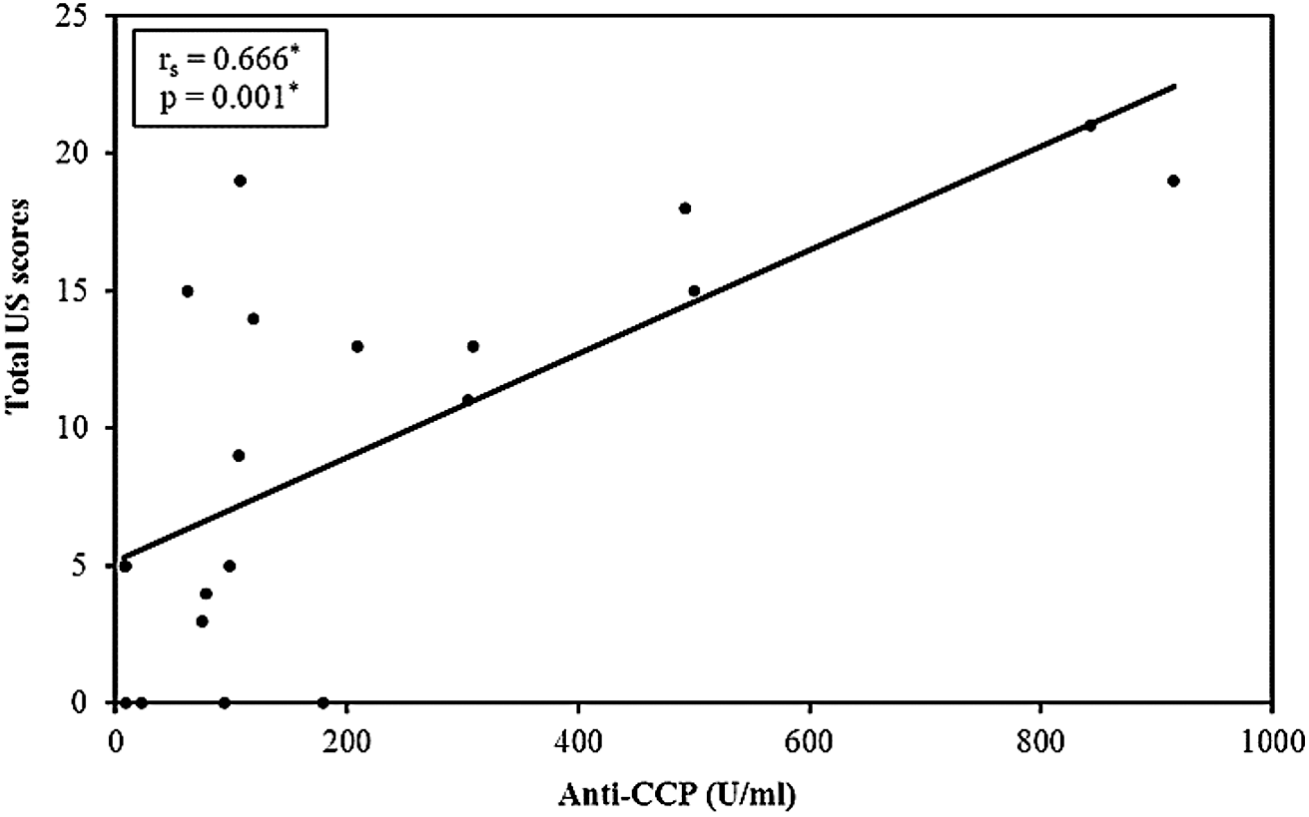
Correlation between total US scores with anti-CCP in RA patients

**Fig. 5:**
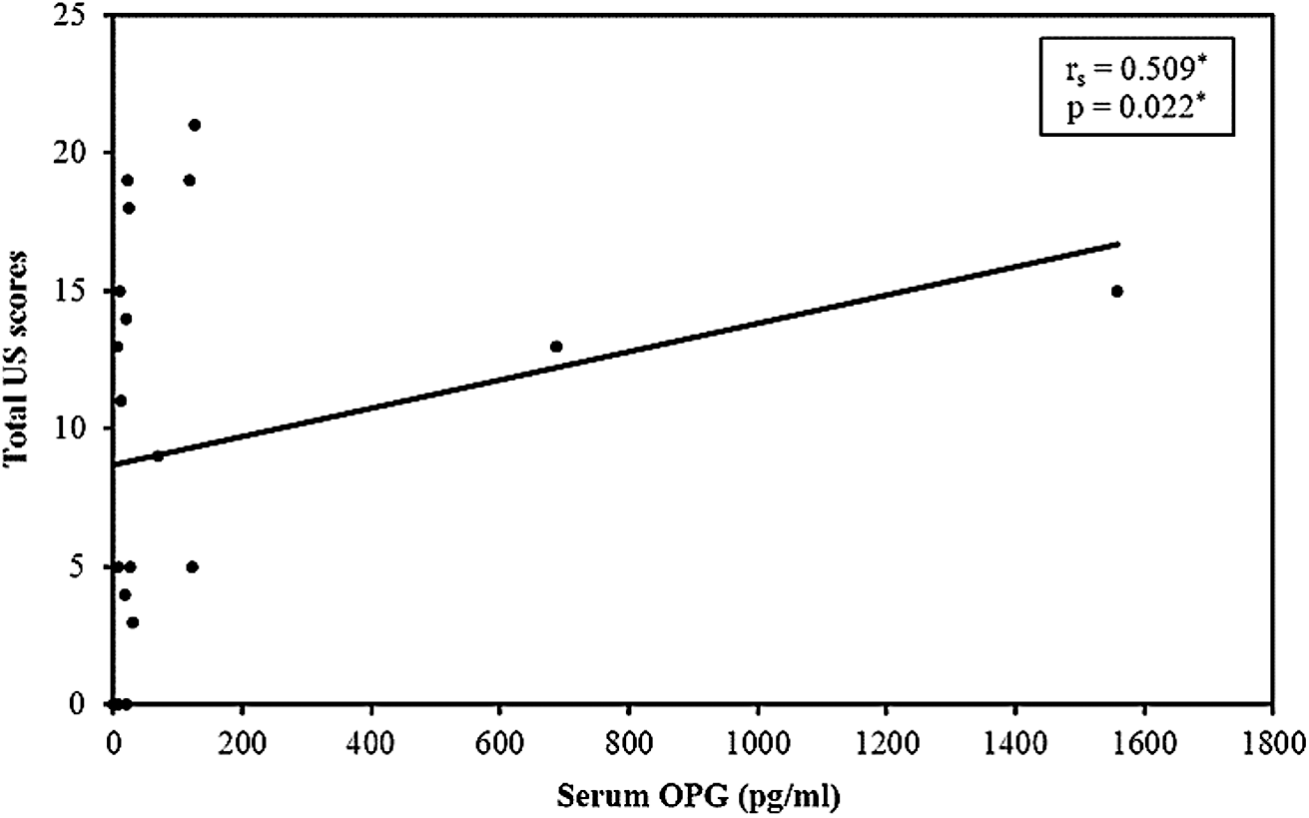
Correlation between total US scores and serum OPG in RA patients

In RA patients, the wrist was most commonly affected on MSUS (*n* = 14; 70%), followed by MCP II and MTP II joints in 11 patients each (55%), MTP V joint in eight (40%), MCP III joint in six (30%), and PIP II joint in four (20%) patients, with least involvement in the PIP III joint in three (15%) RA patients. In FDRs, the wrist was also the most commonly involved joint on MSUS together with MTP II joint in six (24%) FDRs, followed by MCP II joint in one (4%). In FDRs, MCP III, PIP II and III, and MTP V joints were not affected. Correlation between 7-joint ultrasound score and different disease parameters in RA group is shown in (Table 6). **Total US scores showed statistical significant positive correlation with anti-CCP and serum OPG** (Figs. 4 and 5).

**Table 6:** Correlation between 7-joint ultrasound score and different parameters in RA

		US7 score					
		Synovitis GS	Synovitis PD	Tenosynovitis GS	Tenosynovitis PD	US erosions	Total US scores
**Disease duration**	**r**_**s**_	-0.150	-0.089	0.101	0.007	**0.535** ^*^	0.145
	**P**	0.529	0.709	0.671	0.976	**0.015** ^*^	0.542
**DAS28**	**r**_**s**_	0.024	-0.265	0.175	-0.239	-0.077	-0.059
	**P**	0.922	0.259	0.534	0.309	0.747	0.805
**HAQ score**	**r**_**s**_	-0.210	0.005	0.057	-0.013	0.334	0.077
	**P**	0.373	0.982	0.812	0.955	0.151	0.746
**PGA**	**r**_**s**_	-0.349	-0.111	0.208	0.321	-0.082	-0.172
	**P**	0.132	0.640	0.379	0.168	0.731	0.469
**Pain –VAS**	**r**_**s**_	-0.349	-0.111	0.208	0.321	-0.082	-0.172
	**P**	0.132	0.640	0.379	0.168	0.731	0.469
**ESR 1st hr**	**r**_**s**_	0.015	-0.090	-0.087	0.136	-0.336	-0.024
	**P**	0.951	0.707	0.717	0.569	0.147	0.919
**CRP**	**r**_**s**_	-0.241	-0.065	-0.369	-0.306	-0.093	-0.206
	**P**	0.305	0.784	0.110	0.189	0.695	0.385
**RF**	**r**_**s**_	0.176	0.365	**0.525** ^*^	0.346	0.377	0.443
	**P**	0.459	0.114	**0.017** ^*^	0.135	0.101	0.051
**ACPA**	**r**_**s**_	**0.459** ^*^	**0.627** ^*^	**0.675** ^*^	**0.554** ^*^	0.402	**0.666** ^*^
	**P**	**0.042** ^*^	**0.003** ^*^	**0.001** ^*^	**0.011** ^*^	0.079	**0.001** ^*^
**Serum OPN**	**r**_**s**_	0.323	-0.003	-0.092	0.169	0.179	0.342
	**P**	0.165	0.991	0.699	0.475	0.450	0.141
**Serum OPG**	**r**_**s**_	**0.512** ^*^	0.323	0.160	0.136	0.138	**0.509** ^*^
	**P**	**0.021** ^*^	0.165	0.500	0.569	0.562	**0.022** ^*^

rs: Spearman coefficient, *: Statistically significant at *p* ≤ 0.05

US7: 7-joint ultrasound score; GS: Grayscale; PD: power Doppler; DAS: disease activity score; HAQ: health assessment questionnaire; PGA: Patient global assessment; VAS: Visual Analogue Scale; ESR: erythrocyte sedimentation rate; CRP: c-reactive protein; RF: rheumatoid factor; ACPA: anti-citrullinated protein antibodies; OPN: Osteopontin; OPG: Osteoprotegerin

### Plain x-ray findings

Plain x-ray of the scanned hand and foot were performed in all RA patients. Erosions were detected by plain x-ray in eight RA patients. Radiographic erosions were mostly detected in the wrist of five RA (25%) and MCP II joints in five (25%) RA patients, followed by MTP V joint in two (10%) RA patients, then MTP II joint in one (5%) patient. MCP III, PIP II and PIP III joints were not affected by erosions via x-ray.

## Discussion

Biochemical markers of bone and cartilage turnover may provide a potentially sensitive method for detection of active bone and cartilage degradation in RA [29], and it was suggested to reflect similar changes in the pre-clinical phase of RA, thus allowing to identify individuals who are at risk of developing the disease [17]. OPN and OPG are suggested to be related to the metabolic bone changes which occur in RA, but insufficient data are available on their changes in preclinical RA.

There are only two studies in the current literature which measure bone metabolism biomarkers in the preclinical phase of RA, but without definitive conclusions. One study reported the high prevalence of cartilage oligomeric matrix protein in the period near the diagnosis [30]. The other was performed by van Schaardenburg D et al. [17], who reported statistically significant increased mean levels of OPG and N-terminal type I procollagen pro-peptide in preclinical RA compared to controls.

In the current work, we measured serum OPN and OPG in RA and their FDRs compared to healthy controls as markers of pre-clinical disease. OPN levels were higher in RA patients than in controls (without statistically significant difference). OPN levels were lower in FDRs than in controls (also non-significant). Several studies [31–34] reported the finding of significantly raised serum OPN in RA compared to healthy controls. When compared to systemic sclerosis patients, OPN levels were significantly higher in RA [34]. Meanwhile, there was no difference in OPN between systemic sclerosis and controls. As a result of these observations, OPN was suggested to be related to RA disease pathogenesis. Furthermore, a recent meta-analysis reported the significantly raised OPN in RA compared to osteoarthritis patients [35]. The detection of raised OPN in RA compared to other inflammatory and non-inflammatory arthritis strengthens the assumption that it is related to RA pathogenesis.

Although raised OPN levels in RA were confirmed in most studies, its relation to inflammatory parameters were inconsistent. Shaker et al. [31] reported that serum OPN was significantly correlated with parameters of disease activity such as the duration of morning stiffness, ESR and DAS28. Similarly, Bazzichi et al. detected that OPN level was significantly related to CRP and disease severity reflected by the HAQ score in RA patients [34].

In contrast, other studies [32, 33] as well as the current work did not detect a significant correlation between serum OPN in RA patients and DAS28 as a measure of disease activity. In addition, the current work did not reveal any statistically significant correlation between serum OPN and the other assessed parameters in RA patients. This raise the question regards the effect of anti-rheumatic treatments such as NSAIDs, disease-modifying anti-rheumatic drug, and glucocorticoid on the level of OPN. To date, reviewing the literature did not reveal any data about this query and further studies have to focus on the effect of medications on OPN level in RA patients.

The second biochemical markers for bone turnover measured in this study was OPG. Significant differences in OPG levels were detected between the three studied groups, with significantly higher levels in RA patients compared to FDRs and controls. Similarly, most studies reported raised OPG levels in RA compared to controls and correlated it with inflammatory activity in RA [36, 37]. A recent meta-analysis [38] revealed that, compared with the control group, OPG levels were significantly higher in the RA group (*p* < 0.001), and the disease activity score DAS28 was associated with OPG level in RA patients.

In the current study, although there was no correlation between OPG and laboratory parameters of active inflammation (DAS28, ESR and CRP), there was a statistically significant positive correlation between serum OPG and GS synovitis, which reflects joint inflammation. This may support the relation between OPG and inflammation in RA as detected by MSUS.

In order to test the hypothesis that FDRs of RA may have similarly altered bone metabolism, we measured serum OPN and OPG in FDRs without clinical symptoms. All included FDRs were examined by MSUS for assessing the presence of subclinical synovitis; ESR and CRP were also measured. To the best of our knowledge, this is the first study to test serum OPN and OPG in FDRs, also in relation to MSUS.

Mean serum OPN was numerically lower in FDRs than in RA patients and controls. In addition, the mean serum OPG was significantly lower in FDRs than in RA patients. It was numerically higher in FDRs than in controls. There was no significant correlation between serum level of OPN or OPG and different measured parameters in FDRs. The increased OPG in FDRs than controls could reflect an increased osteoclast activity in the preclinical phase, which was previously proposed by van Schaardenburg D et al. [17]. Liu YY et al. suggested that OPG levels vary at different rheumatoid disease stages [39] and found it to be elevated in early RA compared to longstanding disease. Thus, it seems logic to be also raised short before clinical disease onset in pre-RA, consequently to be raised in FDRs than in controls.

The difference in the levels of these markers of bone metabolism in FDRs compared to healthy controls (though, without statistically significant difference) denotes that FDRs possess an alteration from normal bone metabolism. Conforming with the same concept, when multiple cytokines in sera from unaffected FDRs of RA were previously compared to RA and healthy controls, they proved to have distinct cytokine profiles [40]. In order to confirm our findings and to find an explanation for these alterations, testing these biochemical markers on larger scale studies is mandatory.

Eight of 25 (32%) FDRs in the current study had arthralgia, while 17 FDRs (68%) were asymptomatic. This is not unusual, as it is reported that FDRs were significantly more likely to report joint symptoms compared to individuals with no family history of autoimmune disease [41].

In this study, the US7 score was performed for all RA patients (*n* = 20) and their FDRs (*n* = 25).

The detection of MSUS findings in 16/20 (80%) RA patients is an expected finding, but the detection of abnormal MSUS findings in 10/25 (40%) FDRs is noteworthy. Three of 10 (12%) FDRs with MSUS findings had arthralgia and 7/10 (28%) were asymptomatic. Synovitis was the commonest MSUS finding in RA (*n* = 16/20, 80%), and in their FDRs (*n* = 10/25, 40%). In addition, the joints most commonly involved in FDRs were the same as those involved in RA, being the wrist, MTP II and MCP II. Erosions were detected in nine (45%) RA patients and in one (4%) FDR (in MCP II). The high percentage of bone erosions in RA is a further confirmation of the increased osteoclastogenesis. What was not expected is the detection of an erosion in an asymptomatic FDR which could be in favor of the presence of altered bone metabolism that is hypothesized to occur before clinical disease phase.

It was postulated that joint damage is not only the consequence of arthritis, but autoimmune processes that begin years before the clinical disease onset could play a role [3]. Thus, the finding of erosions in asymptomatic FDRs may be a result of an increased osteoclast activity in the preclinical phase which may explain the raised OPG in FDRs compared to controls.

Furthermore, mean ESR and CRP were statistically higher in FDRs than in controls. This conforms to what was previously reported of increased levels of acute phase reactants years before the onset of RA symptoms in blood donors [42, 43]. Of note, the mean ACPA was higher in FDRs than in controls, but without revealing statistical difference. It is well known that RA patients have raised RF and ACPA in the pre-clinical stage. This was first shown in a pivotal study by Nielen et al. [44]. who observed the increased prevalence of autoantibodies over time in serum samples from RA patients collected serially in the preclinical phase before RA becomes clinically evident.

When FDRs with arthralgia were compared to those who were asymptomatic, several observations were done: FDRs with arthralgia had significantly higher ESR and CRP than asymptomatic FDRs (both *p* = 0.003). OPG was higher in those with arthralgia than in those without (though, without statistical difference). Serum OPN was higher in FDRs with arthralgia than in asymptomatic relatives, also without significant difference. Furthermore, mean RF and mean ACPA were higher in FDRs with arthralgia.

Considering the EULAR formulated phases of RA development [45], FDRs of RA with arthralgia could represent the phase of symptoms without clinical arthritis; a more advanced phase in the timeline development of RA than asymptomatic FDRs. Accordingly, this can bring more insight into the pathogenesis of RA development. All these altered findings assume that FDRs already “suffer” subclinical inflammatory, immunologic and imaging alterations in comparison to healthy controls. The results of the current study are consistent with the hypothesis that FDRs are at an increased risk of developing RA than the general population [46], and represent a preclinical phase of RA. Considering this, timely follow-ups of FDRs for early initiation of treatment thus preventing progression to overt disease and disability is mandatory.

### Study limitations

The main limitation of this study is that the number of samples analyzed is relatively small to draw solid and definite conclusions. This was partly due to the difficult access to RA first-degree relatives which is usually occur indirectly through the RA patient themselves. So we can’t recruit all FDR of RA patients but we included some. Future studies are required to include all FDR of RA patients, especially those with environmental risk factors associated with RA with follow up of them over time. Another limitation might be that the US scans were performed by a single assessor though highly experienced in MSUS (ES).

## Conclusions

The elevation of OPN and OPG in RA denotes the already altered bone metabolism. The raised OPN and lower OPG in FDR than controls reflect state of altered bone metabolism. Moreover, FDRs with arthralgia experience higher mean levels of serum OPN, OPG, ESR, CRP, RF, and ACPA than asymptomatic FDRs. These findings reflect an ongoing disturbed bone metabolism and inflammation in FDRs which could precede the clinical disease phase. Thus, OPN and OPG could serve as markers of altered preclinical bone metabolism in rheumatoid FDRs.

The current study suggest the presence of inflammatory synovial changes in FDRs of RA who are free of clinical disease. These findings strengthen the concept of FDRs as pre-RA. While US7 score in RA is significantly correlated to disease duration and autoantibodies, in FDRs US7 score is a useful screening tool to identify subclinical synovitis in at-risk individuals.

More long-term studies on FDRs are needed for establishing the predictive value of abnormal MSUS findings for the development of persistent arthritis. Results need to be confirmed on larger numbers of FDRs for developing criteria for screening symptomatic FDRs for pre-clinical phase of RA and their follow up, aiming at early diagnosis and management to prevent disability. Using MSUS and biomarkers of bone metabolism, in addition to RA autoantibodies, can add to the sensitivity and specificity of the screening methods.

### Electronic supplementary material

Below is the link to the electronic supplementary material.

Supplementary Material 1

## Data Availability

All data generated or analysed during this study are included in this published article [and its supplementary information files].

## Abbreviations

ACPA: Anti-citrullinated protein antibodies
ACR: American college of rheumatology
CRP: C-reactive protein
DAS28: Disease activity score of 28 joints
DMARDs: Disease modifying anti-rheumatic drugs
ESR: Erythrocyte sedimentation rate
EULAR: European League Against Rheumatism
FDRs: First degree relatives
GSUS: Grayscale ultrasound
HAQ: Health assessment questionnaire
MCP: Metacarpophalangeal
MSUS: Musculoskeletal ultrasound
MTP: Metatarsophalangeal
OPG: Osteoprotegerin
OPN: Osteopontin
PDUS: Power Doppler ultrasound
PIP: Proximal interphalangeal
RA: Rheumatoid arthritis
RANKL: Receptor activator of nuclear factor-kappa B ligand
RF: Rheumatoid factor
US7 score: 7-joint ultrasound score
VAS: Visual analogue scales
