# Supplementary material for: Osteopontin, osteoprotegerin and musculoskeletal ultrasound findings in first-degree relatives of rheumatoid arthritis: potential markers of preclinical disease
Source: BMC Musculoskelet Disord. 2024 Mar 5;25:195. doi: 10.1186/s12891-024-07291-7 (PMC10913638; doi:10.1186/s12891-024-07291-7)
Supplement: Supplementary file 1 — Supplementary Material 1 [file 12891_2024_7291_MOESM1_ESM.docx]

**Supplementary Tables**

**Table S1 Correlation between serum OPN and different parameters in the three studied groups**

|  | **Serum OPN** | | | | | |
| --- | --- | --- | --- | --- | --- | --- |
|  | **RA** | | **FDRs** | | **Controls** | |
|  | **r_s_** | **p** | **r_s_** | **p** | **r_s_** | **p** |
| **Disease duration (years)** | 0.104 | 0.662 | - | - | - | - |
| **DAS-28** | 0.063 | 0.791 | - | - | - | - |
| **HAQ score** | 0.338 | 0.145 | - | - | - | - |
| **ESR 1^st^ hr** | 0.058 | 0.807 | 0.217 | 0.297 | 0.012 | 0.973 |
| **CRP** | 0.130 | 0.584 | 0.094 | 0.655 | 0.315 | 0.381 |
| **RF** | 0.222 | 0.346 | 0.306 | 0.137 | 0.313 | 0.379 |
| **ACPA** | 0.133 | 0.576 | 0.057 | 0.785 | 0.207 | 0.567 |
| **Serum OPG** | -0.042 | 0.861 | -0.011 | 0.957 | -0.369 | 0.294 |

***r_s_: Spearman coefficient***

**Table S2 Correlation between serum OPG and different parameters in the three studied groups**

|  | **Serum OPG** | | | | | |
| --- | --- | --- | --- | --- | --- | --- |
|  | **RA** | | **FDRs** | | **Controls** | |
|  | **r_s_** | **p** | **r_s_** | **p** | **r_s_** | **p** |
| **Disease duration (years)** | -0.077 | 0.747 | - | - | - | - |
| **DAS-28** | -0.289 | 0.217 | - | - | - | - |
| **HAQ score** | 0.348 | 0.132 | - | - | - | - |
| **ESR 1^st^ hr** | -0.213 | 0.367 | 0.301 | 0.143 | -0.282 | 0.430 |
| **CRP** | -0.232 | 0.326 | -0.009 | 0.964 | -0.097 | 0.789 |
| **RF** | 0.423 | 0.063 | -0.121 | 0.564 | 0.194 | 0.592 |
| **ACPA** | 0.370 | 0.108 | -0.095 | 0.653 | 0.157 | 0.665 |

***r_s_: Spearman coefficient***
